# Genomic analysis of *Enterococcus faecium* from non-clinical settings: antimicrobial resistance, virulence, and clonal population in livestock and the urban environment

**DOI:** 10.3389/fmicb.2024.1466990

**Published:** 2024-09-11

**Authors:** Jéssica Lopes, Hermínia de Lencastre, Teresa Conceição

**Affiliations:** ^1^Laboratory of Molecular Genetics, Instituto de Tecnologia Química e Biológica António Xavier, Universidade Nova de Lisboa (ITQB NOVA), Oeiras, Portugal; ^2^Laboratory of Microbiology and Infectious Diseases, The Rockefeller University, New York, NY, United States

**Keywords:** *Enterococcus* spp., livestock, environment, *Enterococcus faecium*, antimicrobial resistance, non-clinical enterococcus reservoirs, aminoglycosides high-level resistance, ampicillin resistant *E. faecium*

## Abstract

**Introduction:**

Enterococci are commensals of the gastrointestinal tract of humans and animals that evolved into opportunistic pathogens with high antimicrobial resistance and virulence. Multidrug-resistant *Enterococcus* is a major cause of hospital-acquired infections worldwide. For this reason, the characterization of non-clinical reservoirs of Enterococci and their epidemiological link to resistant hospital isolates is crucial for controlling their spread.

**Methods:**

A total of 295 samples collected from livestock (pigs and cows, *n* = 135) and environment (public buses, passengers hands, and urban environments, *n* = 160) were screened for *Enterococcus* spp. *E. faecium* antimicrobial resistance profiles, virulence potential, and clonal population were further characterized.

**Results:**

Enterococci were detected in 90.5% (*n* = 267) of the samples, with a higher prevalence in livestock (100%) than the environment (82.5%, *p* < 0.0001), but none of the isolates exhibited vancomycin resistance. *E. faecalis* was the most prevalent species (51.7%), predominantly found in livestock (62.2%), while *E. faecium* was more common in the environment. Of the 59 *E. faecium* isolates, 78% showed resistance to ≥3 antibiotic classes and contained associated resistance genes, namely tetracyclines (*tetM* and *tetL*), beta-lactams (mutations in *pbp5*), and high-level resistance to aminoglycosides (*ant(6)-Ia* and *aac(6′)-aph(2″)*). A wide array of virulence factors was detected among *E. faecium*, associated with adherence, biofilm formation, and adaptation to host response, while hospital-associated virulence markers, such as IS16, were less frequent, probably due to the non-clinical nature of the isolates. Clonal population analysis revealed a diverse *E. faecium* population. Although no direct epidemiological link could be traced between our isolates and specific clinical isolates, infection-associated genetic backgrounds were identified in non-clinical isolates: one isolate from pigs belonged to CC17 (ST32), while four isolates belonged to CC94, including one recovered from pigs (ST296), one from cows (ST2206), one from the urban environment (ST1205), and other from buses (ST800).

**Discussion:**

This study underscores a high prevalence of clinically relevant *Enterococcus* species among healthy livestock and the environment. Despite the absence of vancomycin resistance and limited hospital infection-associated clonal lineages, the presence of *E. faecium* with significant virulence potential and resistance to critical antibiotics in human and veterinary medicine highlights the need for continuing surveillance of non-clinical reservoirs.

## 1 Introduction

Enterococci are Gram-positive acid lactic bacteria ubiquitously found in nature in a wide range of habitats, including the gastrointestinal tract (GIT) of humans, vertebrate and invertebrate animals as part of its microbiome, as well as in the natural environment in plants, soil, fresh, and marine waters and in food (Murray, 1990; Byappanahalli et al., 2012; Lebreton et al., 2014; Cattoir, 2022). While the *Enterococcus* genus comprises >50 species, its distribution in nature is highly variable and associated with host species factors such as age and diet or environmental stress conditions (Lebreton et al., 2014; Cattoir, 2022). *Enterococcus faecalis*, *Enterococcus faecium*, *Enterococcus hirae*, and *Enterococcus durans* are the species most commonly found in the gut of mammals, with *E. faecium* and *E. faecalis* representing up to 1% of human adult microbiota (Torres et al., 2018; Cattoir, 2022). Some of these *Enterococcus* species, namely *E. faecalis* and *E. faecium,* evolved as human opportunistic pathogens being responsible for a broad range of infections from the urinary tract, intra-abdominal infections or wounds, to more severe life-threatening conditions such as bacteremia and endocarditis usually associated with high antibiotic resistance and virulence (Cetinkaya et al., 2000; Arias and Murray, 2012; Zhong et al., 2019; Cattoir, 2022; Wei et al., 2024). Although *E. faecalis* accounts for the majority of enterococcal infections in humans, *E. faecium* has been increasingly reported in hospitals usually associated with severe conditions (Gao et al., 2017; ECDC, 2023; Wei et al., 2024). In fact, a major hallmark of enterococci that allowed the success of nosocomial pathogens is its capacity to acquire antimicrobial resistance and adaptation to hostile environments. Enterococci are intrinsically resistant to a variety of antimicrobials and have remarkable genome plasticity that enables the accumulation of mutations and acquisition of external antimicrobial resistance determinants (Cetinkaya et al., 2000; Arias and Murray, 2012; Guzman Prieto et al., 2016).

Of major concern was the emergence of vancomycin-resistant isolates, which through the acquisition of the *van* operon were able to produce altered cell wall peptidoglycan precursors not recognized by glycopeptides (Uttley et al., 1988; Cetinkaya et al., 2000). Vancomycin-resistant *E. faecium* (VRE) become endemic in US hospitals as a consequence of the increasing usage of vancomycin in difficult-to-treat infections, while in European hospitals, VRE infections have been increasing since the COVID-19 pandemics, reaching a mean rate of 17.6% in 2022 (ECDC, 2023). Antibiotic-resistant *E. faecium* is among the top ten bacterial pathogens with the highest antimicrobial resistance-associated mortality, with treatment often restricted to last-resort antibiotics such as linezolid, tigecycline, and daptomycin. However, sporadic-resistant isolates to these antibiotics have been already reported (Bender et al., 2018a), leading VRE to be considered as a high-priority pathogen for drug development by WHO (2024).

Colonized individuals or animals are potential reservoirs of multidrug-resistant *Enterococcus* spp., representing a significant source for pathogen transmission (Garcia-Solache and Rice, 2019; Cattoir, 2022; Wei et al., 2024). As VRE have a high capacity to persist in environmental surfaces and be maintained as commensals among humans and animals in the gut until they achieve optimal conditions to develop infection (Chavers et al., 2003; Zaheer et al., 2020; Wada et al., 2021), the screening of community and environment reservoirs for multidrug-resistant *E. faecium* emerged as an urgent tool to support nosocomial infection control strategies.

In Portugal, VRE infections have been increasingly detected in hospitals paralleling the European scenario (ECDC, 2023). Moreover, healthy individuals without hospital and/or antibiotic exposure were identified as asymptomatic carriers of antibiotic-resistant Enterococci (Novais et al., 2006; Correia et al., 2014; Freitas et al., 2018). While VRE was sporadically detected in food-producing animals and pets (Zaheer et al., 2020; Cattoir, 2022; Gião et al., 2022), a comprehensive screening of environmental reservoirs in the country remains scarce.

Hence, in this study we applied a One Health approach to unveil the major VRE and antibiotic-resistant Enterococci reservoirs among healthy livestock and urban environment in Portugal. We will assess the significance of these reservoirs on the maintenance of hospital-associated multidrug-resistant *Enterococcus*, namely *E. faecium*, clonal lineages, and/or antibiotic resistance and virulence determinants in natural ecosystems, responding to the need for valuable epidemiological information to guide the design of targeted and effective infection control measures.

## 2 Materials and methods

### 2.1 Samples collection

Samples from livestock and environmental sources, previously recovered in the scope of MRSA surveillance studies in Portugal (Conceição et al., 2013, 2017a,b; Lopes et al., 2019), were screened for the presence of vancomycin-resistant enterococci.

A total of 294 samples included in this study were selected in order to include the highest variability and representativeness of collection dates, source, and positivity for other bacterial carriage or contamination (namely MRSA):

#### 2.1.1 Healthy pigs from swine farms

A total of 80 nasal swabs from healthy swine were collected in two independent Portuguese farms producing animals for human consumption, in two screening periods in 2016 and 2018 (Conceição et al., 2017a; Lopes et al., 2019). These farms used amoxicillin (0.5%), colistin (0.5%), and zinc oxide (0.15%) in the feed regimen of all animals until 2016, but since then, one farm banned colistin usage, while the other farm completely eliminated antibiotics, keeping zinc oxide (0.15%) only to prevent gastrointestinal diseases.

#### 2.1.2 Healthy bovines from a cattle farm

A collection of 55 samples (36 swabs from the nasal cavity and 19 from the inguinal region) were recovered from calves and bovines in a beef production herd in the south of Portugal in January 2016 (Conceição et al., 2017b). At the time of sampling, none of the animals presented apparent clinical symptoms of infection and only two cows had been treated with antibiotics (oxytetracycline).

#### 2.1.3 Public buses and hands of public buses’ passengers

Eighty-nine samples recovered from highly touched surfaces in public buses in Lisbon, between May 2011 and May 2012, and eighteen samples of passengers’ hands previously identified as positives for MRSA in May 2012 and in January 2013 were selected for VRE screening (Conceição et al., 2013). Bus sampling was performed before any cleaning procedure in the vehicles allocated to different travel routes, some of them serving hospitals, while the hands of individuals were screened immediately after leaving the buses (Conceição et al., 2013).

#### 2.1.4 Outdoor urban environment

All 52 environmental samples, recovered in June 2013 in the outdoor areas around major hospitals in Lisbon, were included. Samples were previously recovered by swabbing the surfaces of parking meters, stairwells, automated teller machines, bus stops, public phones, and treadmill stops with sterile cotton gauzes humidified with sterile water and further placed into tryptic soy broth (TSB; Becton, Dickinson & Co, New Jersey, United States) tubes and transported to the laboratory. Two samples were found to be contaminated with MRSA.

These non-clinical settings were shown to be reservoirs of opportunistic pathogens such as *S. aureus*/MRSA (Conceição et al., 2013, 2017a,b; Lopes et al., 2019); the samples included in the study were considered as convenience samples, available in the laboratory for the screening of additional bacterial pathogens.

### 2.2 Samples screening, species identification, and *van* genes detection

All samples were previously stored at −80°C at the Laboratory of Molecular Genetics culture collection at ITQB-NOVA. An aliquot (100 μL) of each sample was added to brain heart infusion (BHI; Becton, Dickinson & Co, New Jersey, United States) tubes supplemented with 6 μg/mL of vancomycin and incubated at 37°C with overnight agitation, following the Clinical and Laboratory Standards Institute (CLSI, 2021) guidelines for the detection of vancomycin-resistant enterococcus, except for the usage of broth media to enable a higher inoculum volume to be used directly from the stored samples. Samples were then plated in parallel in non-selective tryptic soy agar (TSA; Becton, Dickinson & Co, New Jersey, United States) plates and chromogenic selective media for *Enterococcus* spp. (Compass *Enterococcus* agar; BIOKAR Diagnosis, Beauvais, France) and for vancomycin-resistant enterococci (CHROMagar VRE; CHROMagar, Paris, France) and incubated at 35 ± 2°C for 24 h. After incubation, plates were visually inspected and presumptive enterococci colonies with distinct morphologies (different sizes, shapes, or colors) were streaked on BHI agar (OXOID, Thermo Fisher Scientific, Maryland, USA) until purity.

*Enterococcus* species were identified by PCR amplification of internal fragments of the species-specific *ddl* gene, for *E. faecium* and *E. faecalis*, and the *sodA* gene, for *E. casseliflavus*, *E. gallinarum*, *E. raffinosus*, *E. hirae,* and *E. durans*, as previously described (Dutka-Malen et al., 1995; Jackson et al., 2004).

The presence of *van* genes (*vanA*, *vanB*, *vanC1,* and *vanC2/3*) was detected by multiplex PCR (Dutka-Malen et al., 1995; Poulsen et al., 1999).

### 2.3 Phenotypic and molecular characterization of *E. faecium* isolates

Given the clinical significance of *E. faecium* carriage and infection in both humans and animals, we performed additional characterization of isolates, resistance mechanisms, and clonal population structure of the *E. faecium* recovered from the different collection samples.

#### 2.3.1 Antibiotic susceptibility testing and detection of linezolid resistance genes

Antimicrobial susceptibility testing was performed for all 59 *E. faecium* isolates through the disk diffusion method for 16 antibiotics: ampicillin (10 μg), chloramphenicol (30 μg), ciprofloxacin (5 μg), erythromycin (15 μg), gentamicin (30 μg), levofloxacin (5 μg), linezolid (10 μg), linezolid (30 μg), nitrofurantoin (100 μg), quinupristin–dalfopristin (15 μg), teicoplanin (30 μg), tetracycline (15 μg), tigecycline (30 μg), trimethoprim (5 μg), trimethoprim-sulfamethoxazole (25 μg), and vancomycin (30 μg), according to the European Committee on Antimicrobial Susceptibility Testing (EUCAST, 2022) guidelines or Clinical and Laboratory Standards Institute (CLSI, 2021) guidelines, whenever EUCAST breakpoints were not available.

Vancomycin and teicoplanin minimal inhibitory concentrations (MIC) were determined by E-test according to manufacturer instructions (bioMérieux, Marcy-l’Étoile, France). Linezolid MIC was assessed by E-test in isolates phenotypically resistant by the disk diffusion method only. Detection of linezolid resistance genes was also performed in a multiplex PCR including primers for *cfr*, *optrA,* and *poxtA* genes (Bender et al., 2019).

Additionally, high-level aminoglycoside resistance (HLAR) was detected by agar dilution method, according to the CLSI guidelines, on BHI agar plates supplemented with gentamicin (500 μg/mL) or streptomycin (2000 μg/mL).

### 2.4 Genomic characterization of *E. faecium* isolates

#### 2.4.1 Whole-genome sequencing (WGS)

A total of 23 *E. faecium* representative isolates were selected for whole genome sequencing (WGS) based on their phenotypic antimicrobial resistance profiles, in order to include the highest variability of resistant patterns concerning the different sources of the isolates in the study (Supplementary Table S1). Total DNA was extracted by the DNeasy Blood and Tissue Kit according to the manufacturer’s protocol (Qiagen, Hilden, Germany). Genomic libraries were created using the Nextera XT DNA Sample Preparation Kit (Illumina, Little Chesterford, United Kingdom) followed by a 150-bp paired-end sequencing with an estimated coverage of 100X. Raw data were assembled *de novo* using the INNUca v3.1 pipeline.^1^ MLST 2^2^ was used to predict *in situ* multilocus sequence type (MLST). Novel identified gene allele sequences or MLST profiles were submitted to the PubMLST website^3^ for new allele or sequence types (ST) assignments, respectively.

#### 2.4.2 Detection of antimicrobial resistance and virulence genetic determinants and plasmids

Genomic data analysis of antimicrobial resistance, virulence, and plasmids was performed in a batch mode using ABRicate^4^ and through the available resources at the Center for Genomic Epidemiology.^5^ To identify antimicrobial resistance genes or mutations responsible for resistant phenotypes, we used ResFinder (Zankari et al., 2012) and Linezolid Resistant Enterococci Finder (LRE-finder) 1.0,^6^ the Comprehensive Antibiotic Resistance Database (CARD) platform (Jia et al., 2017), and the National Center for Biotechnology Information (NCBI AMRFinderPlus).^7^ Virulence determinants were identified through VirulenceFinder 2.0^8^ using the Virulence Factor Database (VFDB) for Enterococcus and the *E. faecium* and *Enterococcus lactis* database recently published (Roer et al., 2024). Detection of bacteriocin determinants was performed through the MyDbFinder 2.0 tool using the enterococcus bacteriocin database described by Tedim et al. (2023). The assessment of *rep* genes for identification of families of plasmids was performed using PlasmidFinder.^9^ All screenings were carried out with default parameters for a minimum identity of 90% and minimum coverage of 60%, except for PlasmidFinder which had a defined default ID threshold of 95%.

#### 2.4.3 *E. faecium* clonal population analysis

Clonal population structure and prevalent clonal lineages were determined based on MLST and on the core genome MLST (cgMLST). cgMLST allele calling was performed using the chewBBACA v2.5 pipeline.^10^ The visualization of relationships between isolates was performed on a minimum spanning tree constructed on PHYLOViZ 2.0.^11^ Isolates with ≤20 cgMLST allele differences were considered clonally related and belonging to the same clonal type (CT) (de Been et al., 2015). Additionally, a distance matrix-based phylogeny was constructed on FastME 2.0 Web server^12^ and the tree was further visualized on Microreact.^13^ A collection of 132 genomes of *E. faecium* infection isolates recovered in Portuguese hospitals between 2019 and 2021 (Conceição et al., 2022) available at the Laboratory of Molecular Genetics (ITQB-NOVA) and BioProject PRJNA1146999 were included for additional analysis and comparison to the livestock and environmental *E. faecium* isolates identified in this study.

### 2.5 Statistical analysis

Nominal categorical variables such as species prevalence, resistance patterns, and sample collections were compared using Fisher’s exact test, with *p*-values <0.05 being considered statistically significant. All statistical analyses were performed using GraphPad Prism software version 9.4 (GraphPad Software, La Jolla California, United States).

### 2.6 Ethics statement

An oral informed consent was obtained at the time of screening of the passengers’ hands for each human participant. Moreover, samples from livestock and environmental sources included in this study were previously recovered in the scope of MRSA surveillance studies in Portugal, which were approved by ethical committees and published. Ethical approval is detailed in several published papers (Conceição et al., 2013, 2017a,b; Lopes et al., 2019). Regarding infection isolates from Portuguese hospitals included in the clonal population analysis, the isolates were recovered as part of the normal clinical routine of each hospital, without impacting the patient’s clinical status. The protocol was approved by the ethics committee of the participating institutions under the approval reference number CA1622.19-3. All ethics statements presented comply with publisher ethics guidelines and policies.

## 3 Results

### 3.1 *Enterococcus* spp. diversity and characterization of vancomycin-resistant genes

**Figure 1:**
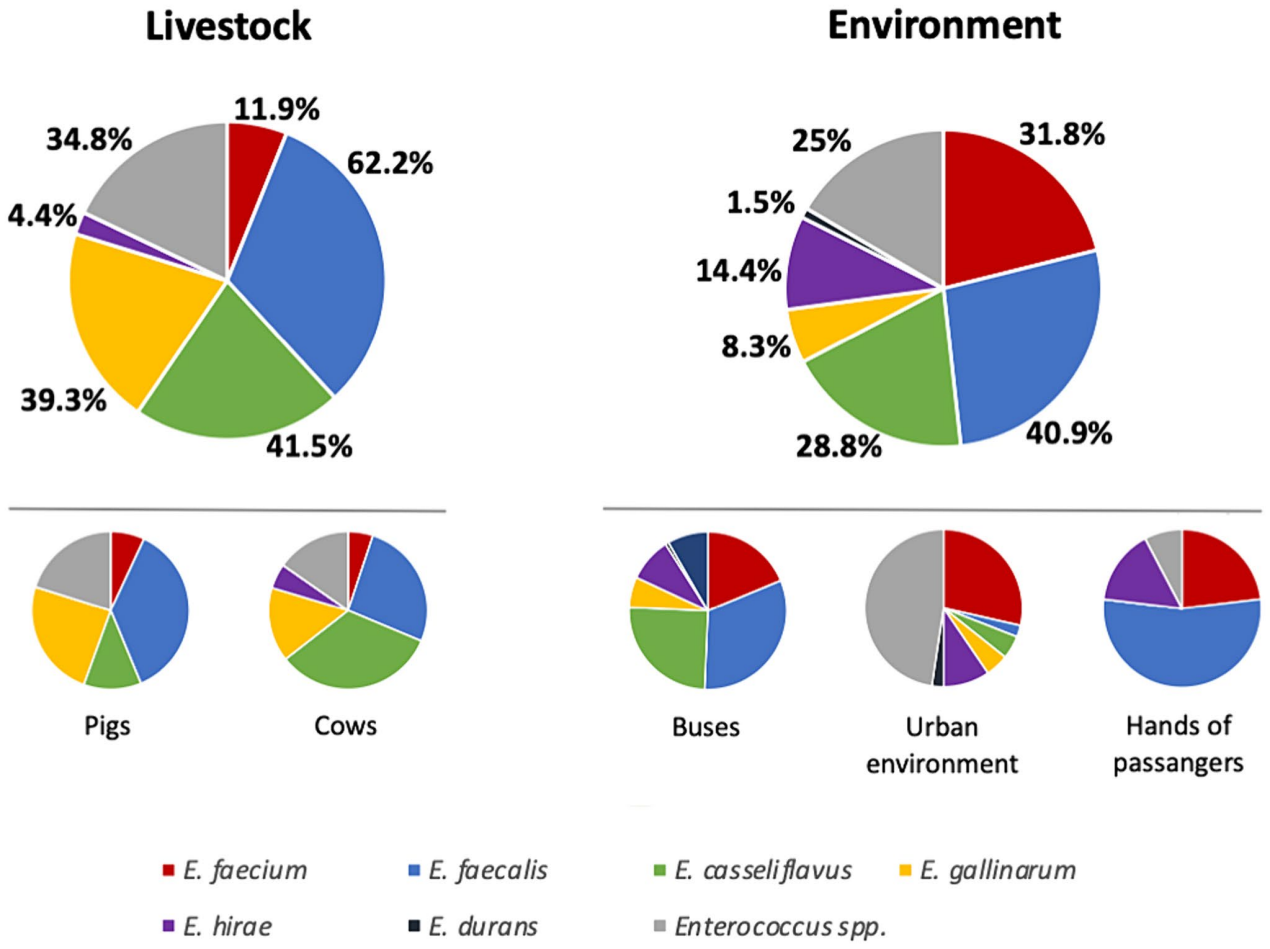
Distribution of enterococcus species by sample collection individually and regarding livestock and environment. Percentages referred to the total of positive samples in each collection.

A total of 295 samples [135 samples from livestock (pigs, *n* = 80; cows *n* = 55) and 160 samples from the environment (public buses, *n* = 90; passenger hands, *n* = 18; urban environment, *n* = 52)] were screened for the presence of enterococci. The overwhelming majority (90.5%, *n* = 267) of the samples contained enterococci, but none of them showed vancomycin resistance. Enterococci were more prevalent among livestock samples (100%) compared to the environment (82.5%) (*p* < 0.0001), namely due to the lower prevalence detected among bus passengers’ hands and urban environment (61% prevalence each). Considering the different species, *E. faecalis* was the most prevalent species detected in half of the enterococcus-positive samples (51.7%), followed by *E. casseliflavus* (35.3%), *E. gallinarum* (24%), *E. faecium* (21.7%), and *E. hirae* (9.4%); *E. durans* was detected in two samples only. While *E. faecalis* seems to be more associated with livestock samples (62.2% versus 40.9%, *p* = 0.0006), being equally detected in pigs (66.3%) and cows (56.4%), *E. faecium* was most prevalent in environment associated samples (31.8% versus 11.9%, *p* < 0.0001) (Figure 1). Moreover, *E. faecalis* and *E. faecium* were the unique species detected in all collections, although at different frequencies (Table 1). *E. casseliflavus* and *E. gallinarum* were not identified among the hands of bus passengers, whereas the two unique *E. durans* isolates were recovered from a bus and a passenger’s hands. *E. raffinosus* was not identified in the study, and other enterococci that could not be classified at the species level (*n* = 84) due to the limited range of species-specific primers available for PCR assays were detected in all collections (Table 1). Buses and the urban environment showed the highest diversity of enterococci, with six different species identified in each collection. Pigs were mostly colonized by *E. faecalis* (66.3%) followed by *E. gallinarum* (43.8%) while the overwhelming majority of cows carried *E. casseliflavus* (70.9%) and *E. faecalis* (56.4%). *E. faecium* was prevalent in the urban environment (37.5%), while the remaining environmental samples were mostly contaminated by *E. faecalis* (Table 1).

**Table 1:** Prevalence of the different *Enterococcus* species and distribution of *van* genes in each sample collection screened.

				*van* gene type (%^c^)			
Sample collection (no. of positives/total of samples)	Species	Number of samples (%^a^)	Number of isolates (%^b^)	*vanA*	*vanB*	*vanC1*	*vanC2/3*
Pigs (*n* = 80/80, 100%)	*E. faecium*	10 (12.5)	11 (7.5)	–	–	–	–
	*E. faecalis*	53 (66.3)	53 (36)	–	–	–	–
	*E. casseliflavus*	17 (21.3)	17 (11.6)	–	–	–	17 (100)
	*E. gallinarum*	35 (43.8)	35 (23.8)	–	–	35 (100)	–
	*E. hirae*	–	–	–	–	–	–
	*E. durans*	–	–	–	–	–	–
	*E. raffinosus*	–	–	–	–	–	–
	*Enterococcus* spp.	29 (36.3)	31 (21.1)	–	–	2 (7)	1 (3)
Cows (*n* = 55/55, 100%)	*E. faecium*	6 (10.9)	6 (4.9)	–	–	–	–
	*E. faecalis*	31 (56.4)	33 (27.3)	–	–	12 (36)	–
	*E. casseliflavus*	39 (70.9)	40 (33.1)	–	–	–	40 (100)
	*E. gallinarum*	18 (32.7)	18 (14.9)	–	–	18 (100)	–
	*E. hirae*	6 (10.9)	6 (4.9)	–	–	–	–
	*E. durans*	–	–	–	–	–	–
	*E. raffinosus*	–	–	–	–	–	–
	*Enterococcus* spp.	18 (32.7)	18 (14.9)	–	–	–	–
Buses (*n* = 89/90, 98.9%)	*E. faecium*	27 (30.3)	27 (18.6)	–	–	–	–
	*E. faecalis*	46 (51.7)	46 (31.7)	–	–	–	–
	*E. casseliflavus*	36 (40.4)	36 (24.8)	–	–	–	36 (100)
	*E. gallinarum*	9 (10.1)	9 (6.2)	–	–	9 (100)	–
	*E. hirae*	13 (14.4)	13 (9)	–	–	–	–
	*E. durans*	1 (1.1)	1 (0.7)	–	–	–	–
	*E. raffinosus*	–	–	–	–	–	–
	*Enterococcus* spp.	12 (13.5)	13 (9)	–	–	1 (8)	7 (54)
Hands of passengers (*n* = 11/18, 61.1%)	*E. faecium*	3 (27.3)	3 (23.1)	–	–	–	–
	*E. faecalis*	7 (63.6)	7 (53.8)	–	–	–	–
	*E. casseliflavus*	–	–	–	–	–	–
	*E. gallinarum*	–	–	–	–	–	–
	*E. hirae*	2 (18.2)	2 (15.4)	–	–	–	–
	*E. durans*	–	–	–	–	–	–
	*E. raffinosus*	–	–	–	–	–	–
	*Enterococcus* spp.	1 (9.1)	1 (7.7)	–	–	–	–
Urban environment (*n* = 32/52, 61.5%)	*E. faecium*	12 (37.5)	12 (27.9)	–	–	–	–
	*E. faecalis*	1 (3.1)	1 (2.3)	–	–	–	–
	*E. casseliflavus*	2 (6.2)	2 (4.7)	–	–	–	2 (100)
	*E. gallinarum*	2 (6.2)	2 (4.7)	–	–	2 (100)	–
	*E. hirae*	4 (12.5)	4 (9.3)	–	–	–	–
	*E. durans*	1 (3.1)	1 (2.3)	–	–	–	–
	*E. raffinosus*	–	–	–	–	–	–
	*Enterococcus* spp.	20 (62.5)	21 (48.8)	–	–	1 (5)	3 (14)
Total collection (*n* = 267/295, 90.5%)	*E. faecium*	58 (21.7)	59 (12.6)	–	–	–	–
	*E. faecalis*	138 (51.7)	140 (29.9)	–	–	12 (9)	–
	*E. casseliflavus*	94 (35.2)	95 (20.3)	–	–	–	95 (100)
	*E. gallinarum*	64 (24)	64 (13.6)	–	–	64 (100)	–
	*E. hirae*	25 (9.4)	25 (5.3)	–	–	–	–
	*E. durans*	2 (0.7)	2 (0.4)	–	–	–	–
	*E. raffinosus*	–	–	–	–	–	–
	*Enterococcus* spp.	80 (30)	84 (17.9)	–	–	4 (5)	11 (13)

a % of species positive samples in the total of positive samples of each collection.

b % of isolates of each species among the total of enterococcus isolates in each sample collection.

c % of isolates carrying the gene in each species per sample collection.

Considering vancomycin-resistant determinants, neither *vanA* nor *vanB* genes were detected. However, *vanC* genes were detected in 40% (186 out of 468) of the isolates, namely *vanC1* detected in 80 (17%) isolates and *vanC2/3* detected in 106 (23%) (Table 1). It is known that *vanC* genes could be intrinsically present in specific enterococcus species, which could be confirmed in our results by the detection of *vanC1* genes in all *E. gallinarum* isolates while *vanC2/3* was exclusively present in all *E. casseliflavus*. Moreover, *vanC1* was additionally detected in 36% of the *E. faecalis* isolated from cows (Table 1), but none of them was identified in samples co-contaminated with *vanC1 E. gallinarum*.

### 3.2 Phenotypic and genomic characterization of *E. faecium* isolates

A total of 59 *E. faecium* isolates (Table 1), recovered from pigs (*n* = 11), cows (*n* = 6), buses (*n* = 27), hands of bus passengers (*n* = 3), and urban environment (*n* = 12), were more thoroughly characterized.

#### 3.2.1 Antibiotic resistance

**Figure 2:**
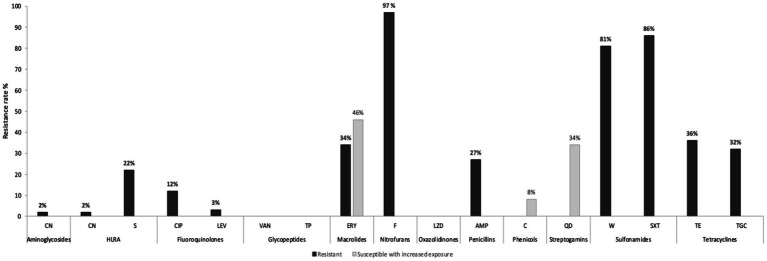
Phenotypic antibiotic resistance profiles of the 59 *E. faecium* isolates. CN, gentamicin; S, streptomycin; HLAR, high-level aminoglycosides resistance; CIP, ciprofloxacin; LEV, levofloxacin; VAN, vancomycin; TC, teicoplanin; ERY, erythromycin; F, nitrofurantoin; LNZ, linezolid; AMP, ampicillin; C, chloramphenicol; QD, quinupristin–dalfopristin; W, trimethoprim; SXT, trimethoprim–sulfamethoxazole; TE, tetracycline; TGC, tigecycline.

The overwhelming majority (78%) of *E. faecium* isolates showed phenotypic resistance to ≥3 antibiotic classes and were considered multidrug-resistant (MDR) (Magiorakos et al., 2012). High resistance rates were related to nitrofurans (nitrofurantoin, 97%), sulfonamides (trimethoprim–sulfamethoxazole, 86%; trimethoprim, 81%), tetracyclines (tetracycline, 36%; tigecycline, 32%), macrolides (erythromycin, 34%), penicillin (ampicillin, 27%), and high-level resistance to aminoglycosides (namely streptomycin, 22%) (Figure 2). None of the isolates showed phenotypic resistance to glycopeptides, namely vancomycin (MICs 0.38–3 μg/mL) and teicoplanin (MICs 0.023–1 μg/mL), nor to linezolid (MIC 1.5–3 μg/mL).

MDR phenotype was highly detected in livestock isolates (94%) compared to environment samples (71%) although this difference was not statistically significant (*p* = 0.0838). On the other hand, tetracycline and ampicillin resistances were significantly associated with livestock. Tetracycline was detected in 64.7% of livestock isolates versus 23.8% in the environment (*p* = 0.0059), while ampicillin was identified in 52.9% of animal isolates (pigs *n* = 7; cows *n* = 2) compared to 17% of the environmental *E. faecium* (*p* = 0.0085). Globally, high-level resistance (HLR) to gentamycin was detected in 2% of *E. faecium*, while 22% showed HLR to streptomycin, mainly associated with livestock isolates (90%) (*p* = 0.0002).

**Figure 3:**
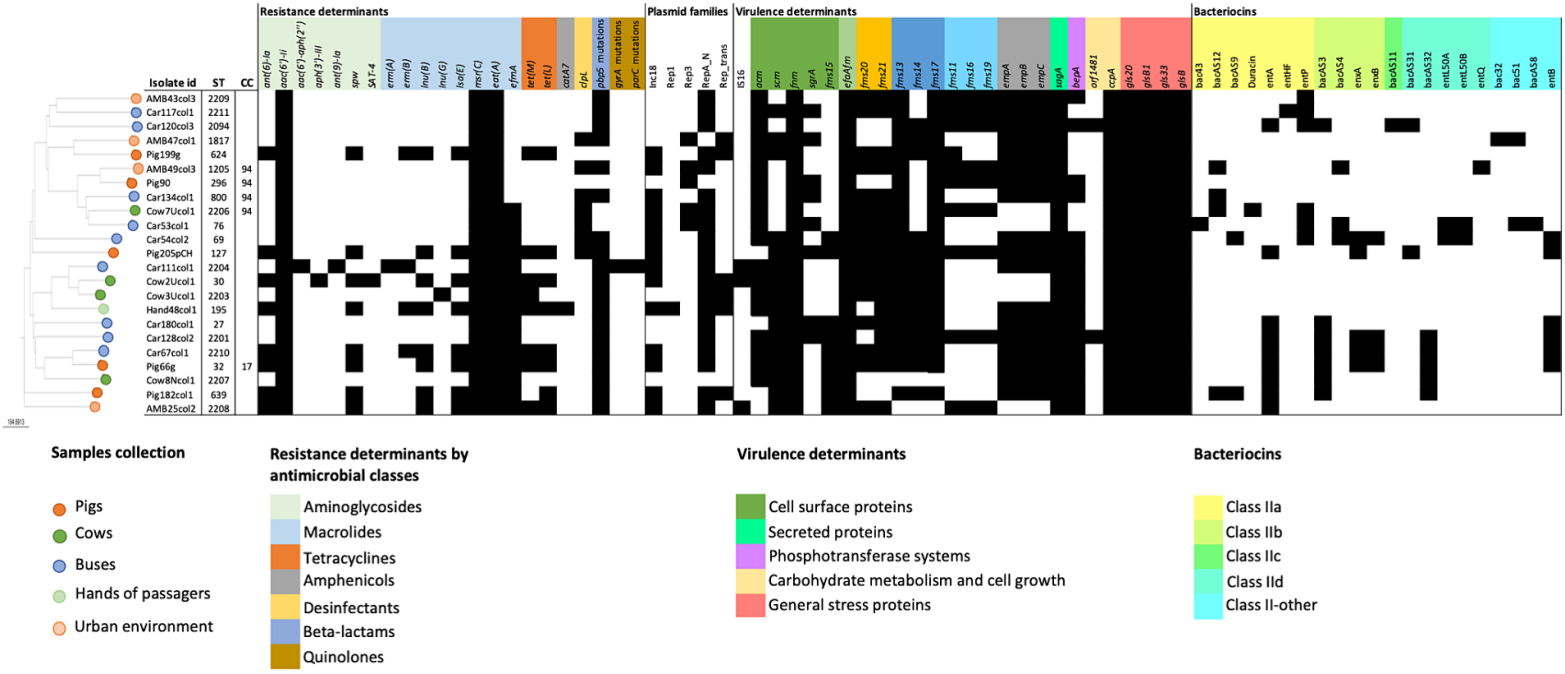
Genomic data analysis regarding cgMLST distance-based matrix phylogeny, antimicrobial resistance, virulence, and plasmids of the 23 *E. faecium* selected for WGS. Antimicrobial resistance genes and mutations (*vanA*, *vanB*, *cfr*, *optrA,* or *poxtA* and 23S rRNA mutations) or virulence determinants (*esp., ptsD*, *hylfm*, *cylA,* or *gelE*) not detected in any isolate were not included. Dark full squares indicate the presence of the gene or mutation; ST, sequence type; CC, clonal complex.

Genomic characterization of 23 selected *E. faecium* isolates allowed the identification of resistance genes associated with MDR phenotype in 87% (20/23) of the isolates (Figure 3). A single isolate, recovered from the hands of a bus passenger, contained resistance genes associated with five antimicrobial classes (aminoglycosides, amphenicols, β-lactam, macrolides, and tetracyclines).

None of the *E. faecium* isolates contained vancomycin-resistant genes, supporting the previous PCR and phenotypic antimicrobial susceptibility results. Moreover, linezolid phenotypic susceptibility was confirmed by the non-detection of resistance genes (*cfr*, *optrA,* and *poxtA*) nor 23S rRNA mutations.

Low-level resistance to aminoglycosides could be attributed to the ubiquitous presence of the aminoglycoside 6′ acetyltransferase [AAC(6′)-Ii] in this species, which was confirmed by the detection of *aac(6′)-Ii* genetic determinant in all *E. faecium* isolates in this study. On the other hand, the genomic analysis showed that the phenotypically detected high-level aminoglycoside resistance was mostly associated with the presence of the *ant(6)-la* gene in the case of streptomycin and to *aac(6′)-aph(2″)* gene in the single high-level gentamicin-resistant isolate (Figure 3). Other aminoglycoside resistance-associated genes were also detected, but at a lower frequency as *spw* (35%), *aph(3′)-IIIa*, *sat-4,* and *ant(9)-la* with 4% each.

Regarding resistance determinants of macrolides and lincosamides, in addition to *msr(C)* that was intrinsically present in all *E. faecium* isolates, other genes, such as *efmA*, *lnu(B)*, *lnu(G), lsa(E)*, *erm(B),* and *erm(A),* were also detected in the sampled collections, although at different percentages (Figure 3).

Resistance to β-lactams, namely ampicillin, was mainly conferred by mutations in the *pbp5* gene that were detected in 83% of the isolates (*n* = 19) (Figure 3), varying from 2 to 18 mutations per isolate. Seven isolates (from pigs, cows, urban environments, and buses) contained the *clpL* gene, commonly associated with decreased penicillin susceptibility and to thermoresistant phenotypes. In addition, 39% (8/23) of *E. faecium* harbored tetracycline resistance genes *tet(M)* and/or *tet(L)*, including two isolates phenotypically resistant also to tigecycline. The genes *tet(M)* and *tet(L)* are commonly associated with tetracycline-resistant enterococcus from animals, which is in accordance with the high tetracycline resistance prevalence of 73% among pigs and 50% among cows observed in this study.

*E. faecium* isolated from buses showed a higher diversity of resistance genes (*n* = 15), while the single sequenced *E. faecium* from the hands of a passenger combined the higher number of resistance genes (*n* = 12), including the *catA7* gene associated with phenotypic resistance to chloramphenicol.

#### 3.2.2 Virulence determinants and bacteriocins

A high prevalence of putative virulence markers was detected among *E. faecium* isolates from all collections studied, including 18 out of the 32 tested determinants identified in >70% of the isolates (Figure 3). Almost half of the isolates (43%) contained ≥20 virulence markers, mostly environmental isolates, 57% versus 33% of livestock, although the difference was not significant (*p* = 0.1068). Cell surface proteins associated with bacterial adherence including adhesins genes *efaA_fm_*, *fnm* (100% each), *acm* (83%), and pili-associated proteins *fms13* and *fms17* (91% each) were identified in more than 80% of the isolates. All isolates (100%) contained determinants involved in biofilm production and cell growth such as *sagA*, a peptidoglycan secreted hydrolase, and *ccpA,* a catabolite control protein A, as well as *gls 20*-*glsB* and *gls 33*-*glsB1* general stress response regulators influencing colonization and adaptation to host response.

On the other hand, virulence determinants with proven impact in biofilm and endocarditis infection such as *empA*/*empB*/*empC* cluster (78%) or *bepA* (74%) were widely detected.

Three isolates, from the urban environment, a bus, and a cow sample, contained the insertion sequence 16 (IS16), a marker associated with hospital strains that contribute to *E. faecium* genomic plasticity and host adaptation. Other virulence determinants associated with clinical isolates or antibiotic-resistant strains as *esp., ptsD*, *hylfm*, *cylA,* or *gelE* were not detected in our collections (Figure 3).

Genes associated with bacteriocins were detected in 78% of the *E. faecium* isolates. Environmental isolates showed a high variability of bacteriocin genes (*n* = 20), and a high prevalence of bacteriocin *E. faecium* producers (*n* = 12.86%) compared to livestock isolates (*n* = 11 different bacteriocin genes and 67% (*n* = 6) bacteriocin producers). Differences were observed regarding the variability of bacteriocin gene content among the different collections, with bus isolates containing 18 different bacteriocin genes compared to urban environments (*n* = 6), pigs (*n* = 9), and cows (*n* = 7) (Figure 3). Moreover, bus isolates showed also the highest bacteriocin content per isolate, with Car54 and Car53 harboring, respectively, 8 and 7 bacteriocin genes. None of the tested bacteriocins could be detected among the isolate of hands. Globally, the most prevalent toxins detected in ≥25% of the isolates included enterocin A (*entA*, 39%), enterocin B (*entB*, 26%), enterocin P (*entP*, 26%), and bacAS3 (30%). Among these bacteriocins, *entA* was the unique gene detected in all studied collections, although at different frequencies. The gene *entB* was present in bus and isolates of the pig while *entP* was found in all except isolates of pigs. The gene *bacAS3* was not detected among urban environmental isolates.

#### 3.2.3 Plasmidome

Globally, the *in silico* analysis identified a high diversity of plasmids among the *E. faecium* genomes. Plasmids from RepA_N and Inc18 *rep* families were the most prevalent, detected in 86.9 and 60.8% of the isolates, respectively, and were present in all five sample collections (Figure 3). No major associations were detected between *rep* genes and specific resistance or virulence determinants in our *E. faecium* isolates, except for the Rep_1 plasmid found in the unique isolate from the hands of a passenger. Rep_1 was probably associated with chloramphenicol resistance detected in this isolate, given the co-localization of *rep* and the *catA7* gene.

#### 3.2.4 Clonal population structure

Clonal population analysis showed a highly diverse *E. faecium* population structure. Each isolate was characterized by a different sequence type (ST), including eight new STs (ST2203, ST2204, ST2206, ST2207, ST2208, ST2209, ST2210, and ST2211) (Figure 3). Five STs could be assigned to two major *E. faecium* clonal complexes (CC): ST32 which belonged to CC17 and was identified in a single isolate from a pig; and ST296, ST2206, ST1205, and ST800, belonging to CC94 and recovered from different sources (one from pigs, one from cows, one from the urban environment, and other from buses). The remaining 18 isolates were considered as single STs. A more detailed cgMLST analysis confirmed the high variability in the population with all cgMLST profiles showing more than 20 allele differences between them and therefore were assigned to different clonal types (CT) (de Been et al., 2015).

**Figure 4:**
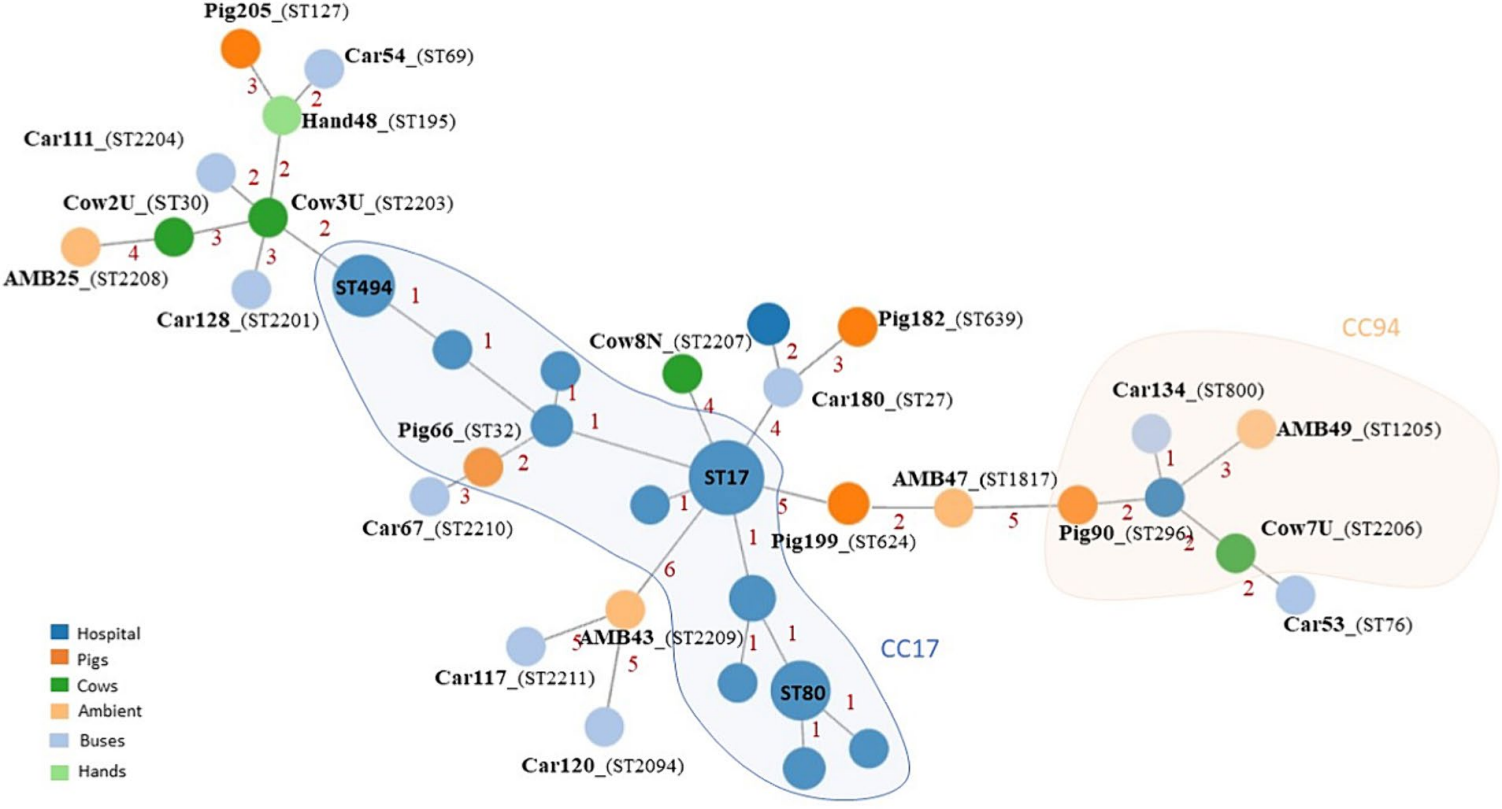
Minimum spanning tree based on MLST analysis of 23 *E. faecium* isolates from livestock and environment and *E. faecium* isolated from infections in Portuguese hospitals between 2019 and 2020. AMB, Urban environment; representative isolates of *E. faecium* derived from this study are labeled in boldface.

Clonal population analysis, including genomic data of *E. faecium* isolated from infection in Portuguese hospitals, showed that none of the *E. faecium* isolates from this study and clinical isolates shared the same ST (Figure 4; Supplementary Tables S1, S2). However, a few related STs could be identified between both collections, namely related to CC94: ST800 identified in a bus isolate and ST1205 isolated in the environment were, respectively, single-locus and triple-locus variants of ST94; ST296 from a pig is a double-locus variant of the clinical ST2206. Two livestock isolates characterized by ST32 and ST2203, belonged to CC17 and were highly related to ST17 and ST494, the two most prevalent STs among clinical *E. faecium* in Portugal between 2019 and 2020. Moreover, isolate Car180col1 shared six out of the seven MLST alleles with a clinical ST2020 isolate.

On the other hand, based on the 1,159 loci common to all *E. faecium* isolates, cgMLST analysis confirmed a high population diversity between isolates identified in this study and the hospital-associated population. None of the isolates from both collections belonged to common CTs, differing between 377 and 1,082 allelic differences. Therefore, no direct epidemiological link between *E. faecium* derived from this study and the *E. faecium* recovered from infection in Portuguese hospitals could be drawn, when considering a more discriminatory method as cgMLST.

## 4 Discussion

This study aimed at the identification of enterococci reservoirs outside hospitals, namely healthy livestock or environmental surfaces, providing valuable knowledge on the dissemination and resistance mechanisms of this major human pathogen. To the best of our knowledge, our study represents the first surveillance of non-clinical settings as the hands of passengers and outdoor urban surfaces near hospitals as possible reservoirs for VRE. Still, this is the first study in Portugal to explore livestock nares and udder regions as potential VRE colonization reservoirs.

In this study, *E. faecalis* and *E. faecium* were the unique species identified in all collections, although at different percentages depending on the sample source. Our results are in line with a recent One Health continuum surveillance of *Enterococcus* spp. that reveals *E. faecium* and *E. faecalis* as the predominant species related to human-associated environments (Zaheer et al., 2020). In fact, buses and hands of passengers were highly contaminated with *E. faecalis* and *E. faecium,* confirming that community settings could be reservoirs of major human pathogens, as previously shown for methicillin-resistant *Staphylococcus aureus* (Conceição et al., 2013). A similar study in Turkey supported also the role of public transports as reservoirs of *Enterococcus* spp.; however, isolates were not identified at the species level (Birteksöz Tan and Erdogdu, 2017). Moreover, we identified clinically relevant *Enterococcus* spp. in other urban environmental public surfaces highly touched by the hands of individuals, as previously identified in shared urban bicycles in China (Gu et al., 2020). As we screened urban environmental surfaces close to hospitals, and it is known that individuals with direct contact with the nosocomial environment have a high probability of having hands contaminated by VRE and transmitting these pathogens to the hospitals’ outside environment (Hayden et al., 2008), our study highlights the importance of hands’ hygiene measures to prevent human pathogens dissemination.

In our livestock samples, *E. faecalis* was the most prevalent species detected, as recently reported in pigs and to a lesser extent in cows cecal swabs collected at slaughterhouses in Portugal (Gião et al., 2022). Moreover, contrary to other studies that showed *E. hirae* as a prevalent species colonizing the gut of cattle and further detected in the food production chain (Zaheer et al., 2020; Gião et al., 2022), in our collection *E. hirae* was randomly detected comparing to the predominance of *E. casseliflavus*. We swabbed the nares and the udder region of cattle animals, which could justify this difference among the enterococcus species identified.

Pigs were highly nasally colonized by *Enterococcus* spp., namely *E. faecalis* has recently been reported in four pig farms in Spain (Abdullahi et al., 2023), which suggest that although the nares are not the common habitat for *Enterococcus* species, nasal sampling might be valuable for enterococci monitoring in animals.

Vancomycin resistance was not detected in our samples. Although isolates were able to initially grow in media supplemented with 6 μg/mL of vancomycin, none of them grew in VRE-selective media. The presence of other bacteria in our initial multibacterial samples might produce factors that protect enterococci from vancomycin or alter the local environment in a way that reduces the effective concentration of the antibiotic to concentrations that allow the growth of susceptible isolates, or the low-level resistance to vancomycin phenotype intrinsic of some enterococcus species could support this observation. VRE has already been identified in animals in Portugal (Freitas et al., 2009; Ramos et al., 2012; Braga et al., 2013; Novais et al., 2013), but there are no data regarding VRE in non-clinical environmental settings. Our study contributes to fulfilling this gap, identifying multidrug-resistant *E. faecium* isolates in buses, passengers, and urban environments, in addition to vancomycin resistance.

The *vanC* genes are considered to be intrinsic to *E. gallinarum* (*vanC1*), *E. casseliflavus* (*vanC2*), and *E. flavescens* (*vanC3*) (Ahmed and Baptiste, 2018; Torres et al., 2018). However, *vanC1* was detected in 36% of our *E. faecalis* isolates from cows, which is in line with the increasing reports of *vanC1* in *E. faecalis* from animal-associated environment samples (Schwaiger et al., 2012; de Garnica et al., 2013; Nishiyama et al., 2015). On the other hand, none of the *E. faecium* isolated in our study harbor *van* genes, although *vanC1* was already reported in *E. faecium* from human infection (Sun et al., 2014), evidencing that *vanC1* could be spread to other clinically relevant Enterococcus species. Although a possible bias toward the selection of intrinsic vancomycin-resistant enterococcus could be suggested by the usage of 6 μg/mL of vancomycin in the first screening of samples, no other *van* genes or vancomycin non-susceptibility could be detected in the majority of the Enterococcus isolates in addition to naturally intrinsic resistant *E. casseliflavus* and *E. gallinarum* and 9% of the total *E. faecalis* isolates.

Given the clinical relevance of *E. faecium* in human infection, namely VRE that is considered a high-priority pathogen by the WHO (2024), a comprehensive characterization of enterococci isolated in the present study was focused on *E. faecium* isolates.

The rationale to further study the *E. faecium* isolates was supported by a significant increase of *E. faecium* in nosocomial infections worldwide, namely in Europe, associated not only with vancomycin resistance but also with other antibiotics used in human clinical practice and in veterinary medicine as ampicillin, aminoglycosides (high-level resistance), or last-resort antibiotics like linezolid and tigecycline. In the most recent Antimicrobial Surveillance Report 2022, the ECDC reported a significantly increasing trend of antimicrobial-resistant *E. faecium* in some individual countries in Europe and highlighted the urgent need for close monitoring to better understand the epidemiology, clonal diversity, and risk factors associated with vancomycin-resistant *E. faecium* infections (ECDC, 2023). In Portugal, the country where the study was carried out, VRE has been increasingly detected in hospitals compared to a decreasing trend observed in *E. faecalis* resistant to aminoglycosides since 2018, which heightened concern among hospitals about these resistant *E. faecium* infections and the need for monitoring reservoirs and the development of effective infection control guidelines (ECDC, 2023). Additionally, *E. faecium* reservoirs have been identified in a One Health continuum (Zaheer et al., 2020), suggesting that this relevant species for human health, may be prevalent in non-clinical environments and could potentially be transmitted to humans.

We identified high rates of antimicrobial resistance among the isolates independently of the source. Resistance of ampicillin and tetracycline was significantly associated with livestock samples, which could be a consequence of the usage of a β-lactam (amoxicillin) as prophylaxis in the feed regimen of pigs at the time of sampling, or with the global and widespread use of tetracycline for prophylactic purposes in livestock (Roberts and Schwarz, 2016). The gene *tet(M)* is considered the most frequent mechanism of tetracycline resistance in enterococci from humans, animals, food, and the environment (Cho et al., 2020), even though in this study *tet(M)* and *tet(L)* were equally detected. In addition, 32% of *E. faecium* from non-clinical settings presented phenotypic resistance to tigecycline, but only two isolates harbored *tet(L)* and *tet(M)*, suggesting that other resistance mechanisms could be involved that deserve further investigation.

The ampicillin resistance rate of 56% among livestock in our study surpasses the 30% resistance reported in poultry *E. faecium* in Portugal (Poeta et al., 2006), but parallels the increasing rates of vancomycin-susceptible ampicillin-resistant *E. faecium* causing infections in European hospitals (Garcia-Solache and Rice, 2019). On the other hand, the significant use of aminoglycosides in veterinary medicine reflects the rates of high-level aminoglycosides resistance in *E. faecium* from livestock and human isolates (McGlinchey et al., 2008; Li et al., 2015; Diab et al., 2019). We detected approximately 24% HLAR in *E. faecium* isolates from non-clinical settings, associated with the presence of the *ant(6)-la* gene, as previously reported in animal *E. faecium* isolates (Han et al., 2011; El-Mahdy et al., 2018; Cagnoli et al., 2022) and suggesting a livestock reservoir for this acetyltransferase gene. In 2022, 34.9% of *E. faecium* isolated from invasive infections in Europe showed aminopenicillins and gentamicin high-level resistance (ECDC, 2023). We identified non-clinical environments and healthy livestock as reservoirs of ampicillin and high-level aminoglycosides-resistant *E. faecium* in Portugal. This finding is of major concern as the spread of these resistant isolates compromises the clinical utility of the commonly used synergistic protocol for aminoglycosides with a cell wall-active antibiotic such as ampicillin in the treatment of severe enterococcal infections (Miller et al., 2014; Echeverria-Esnal et al., 2023).

Moreover, in our study, almost all isolates presented phenotypic resistance to nitrofurantoin, a synthetic antibiotic used as the first-line therapy of uncomplicated lower urinary tract infection caused by HLAR *Enterococcus* and VRE (Mahdizade Ari et al., 2023). High nitrofurantoin resistance rates (56–84%) have been reported in clinical *E. faecium* isolates (Wang et al., 2013), but the identification of such a high prevalence of resistance in non-clinical isolates in our study is of major concern.

In our collections, *E. faecium* harbored an arsenal of virulence determinants associated with bacterial adherence and biofilm production such as *efaA_fm_*, *fnm*, *acm,* and pili-associated proteins, which provide them an advantage on environmental persistence. Moreover, adhesion is usually the first step required for infection, suggesting that these environmental surfaces and healthy livestock represent reservoirs of potentially high pathogenic *E. faecium* isolates. This assumption is even supported by the high prevalence of virulence determinants with proven impact in biofilm and endocarditis infection as *empA*/*empB*/*empC* cluster, *bepA* or *efaA*_fm_, widespread in *E. faecium* isolates, regardless of the isolation source. Putative virulence markers for *E. faecium* hospital-associated strains, such as insertion sequence 16 (IS16) or *acm* and *scm* adhesins (Werner et al., 2011), were also widely detected supporting the pathogenic potential of these environmental and livestock-associated *E. faecium* and showed a different scenario previously reported across a One Health continuum in Canada where livestock-associated *Enterococcus* spp. lacked virulence genes associated with clinical isolates (Zaheer et al., 2020). In addition, three genes (*sgrA*, *fms15,* and *orf1481*) exclusively associated with *E. faecium* hospital variants (Roer et al., 2024) were detected among *E. faecium* in this study, namely in bus isolates, suggesting a possible hospital-associated origin and dissemination to the community.

Bacteriocin production ensures niche competition of enterococci in the gut, namely against particular pathogens or adaptation to different environmental conditions (Tedim et al., 2023). In fact, in our collection, environmental isolates harbored a higher bacteriocin genetic determinants content, belonging exclusively to the heat-stable, non-lantibiotic class II bacteriocins. *entA,* a pediocin-like bacteriocin (class IIa) with strong antilisterial effects, and *bac*AS3 (subclass Iib) were the most prevalent bacteriocin determinants identified in our study (39 and 30%, respectively) as previously reported in a large and diverse collection of *E. faecium* isolates (Tedim et al., 2023). Enterocin gene *entA* was already identified not only in food-associated *E. faecium* but also in clinical outbreaks of VRE- and vancomycin-susceptible *E. faecium* in different European hospitals (Freitas et al., 2016). Moreover, 22% of our isolates, mainly isolates from the bus, contained >5 different *bac* genes, which is much higher than the previously *bac* mean content (1 to 5) among *E. faecium* (Tedim et al., 2023), suggesting a high adaptive potential of our environmental isolates.

Population analysis of the 23 *E. faecium* representative isolates showed a highly polyclonal bacterial collection, regardless of the isolation source. This variability in clonal population structure was not surprising given the non-clinical nature of these *E. faecium* isolates. Moreover, the identification of new STs, namely in cows (ST2203, ST2206, and ST2207), buses (ST2204, ST2210, and ST2211), and urban environmental surfaces (ST2208 and ST2209) suggests the acquisition of genetic variability to improve adaptation and maintenance in adverse environmental conditions or hosts.

Although cgMLST analysis could not trace a direct transmission link between *E. faecium* from invasive infections in Portuguese hospitals and our non-clinical *E. faecium* isolates, the genetic background of several isolates from pigs (ST32-CC17; ST624; ST127; ST296), cows (ST30; ST2206-CC94), buses (ST27, ST2201; ST800-CC94), and urban environment (ST1205-CC94) were already identified in human clinical infections and carriage (see text footnote 3) (Werner et al., 2012; Bender et al., 2018b; Zhou et al., 2019; Peng et al., 2022). In line with our observation, recent studies suggested that instead of an evident gene flow from livestock *E. faecium* to hospital *E. faecium*, it is supported by distinctive niche adaptations for the distribution of antimicrobial resistance genes, host adaptation, and virulence determinants among *E. faecium* population (Gouliouris et al., 2018; Arredondo-Alonso et al., 2020).

The identification in our study of *E. faecium* isolates belonging to genetic backgrounds prevalent in infection and associated with antimicrobial resistance to last-resort antibiotics is a relevant finding. Therefore, ST32 belonged to the CC17, the major hospital-associated clonal lineage responsible for human infections worldwide. While ST32 was detected in a pig nasal swab, it was already identified from carriage and infection in other livestock and humans as well as from environmental wastewaters (see text footnote 3) (Werner et al., 2012). Recent findings from Portugal reported linezolid-resistant ST32 *E. faecium* in a healthy human rectal swab (Freitas et al., 2020), highlighting its significant adaptive and pathogenic potential.

On the other hand, ST30 identified in our study in a cow sample was recently reported in German hospitals containing the *optrA* linezolid resistance gene (Bender et al., 2018b). In Portugal, ST30 *E. faecium* isolates were reported in a pig, dust, and food of pigs in the same farm (Novais et al., 2013). Our detection of ST30 in a cow sample underscores its capacity for host diversification.

CC94 *E. faecium* lineage is mostly associated with human community and commensal isolates that can cause sporadic infections in animals and humans (Peng et al., 2022) In our study, single isolates from livestock (a pigs ST296 isolate and a cow ST2206), a bus (ST800), and urban environment (ST1205) exhibited loci variations of the ST94, indicating their affiliation with the widespread CC94 lineage.

*E. faecium* isolated in this study and the *E. faecium* recovered from infection in Portuguese hospitals differed in the isolation period, which could be a limitation in the genomic comparison analysis. In fact, a more global approach as MLST identified isolates in this study sharing the same genetic background as infection isolates from hospitals, but the high discriminatory power of cgMLST could not identify a direct link between these isolates, which could be due to evolutionary pressure that allows the accumulation of small genetic events along time, promoting adaptation to selective pressure of each environment. An example to support this assumption was the identification in this study of an *E. faecium* bus isolate sampled in 2012 and characterized by ST27 that belonged to the same genetic background (sharing five MLST alleles) of an ST2020 isolate causing infection in hospitals in the same geographic area approximately 8 years later.

Another possible limitation of the study was the inclusion of livestock nasal swabs, which is not the primary niche of *Enterococcus* colonization in humans and animals. However, it has been shown that *Enterococcus* can be found in colonization of the anterior nares and co-habit with other pathogens such as *S. aureus*. Therefore, the previous identification of these samples as reservoirs of *S. aureus* and MRSA (Conceição et al., 2013, 2017a,b; Lopes et al., 2019) as well as the close living conditions of the animals (namely pigs, that live approximately 32 to 33 animals in 8–12 m^2^ stockyards) made the samples to be considered potential reservoirs for multidrug-resistant VRE and therefore selected for this study.

Major achievements of this study included the unveiling of a global high prevalence of Enterococcus spp. in non-clinical settings such as livestock- and human-associated environments, including clinically relevant species such as *E. faecium* and *E. faecalis*. Although no vancomycin resistance could be detected, livestock and environmental surfaces showed to be reservoirs of *E. faecium* isolates with antimicrobial resistance to clinically relevant antibiotics, namely aminoglycosides (high-level resistance), ampicillin and tetracyclines including tigecycline. Moreover, these *E. faecium* isolates harbor a wide arsenal of virulence determinants associated with increased adaptation to hostile environments and different hosts, evasion of the immune system, and capacity of infection development, associated with genetic backgrounds already identified in infection isolates. Our results evidenced that surveillance of *Enterococcus* spp., with a special focus on *E. faecium* outside nosocomial settings is of paramount importance for effective infection control strategies and reduction of antimicrobial resistance spread, given its proven role as reservoirs of multidrug resistance and virulent strains.

## Data Availability

The datasets presented in this study can be found in online repositories. The names of the repository/repositories and accession number(s) can be found in the article/Supplementary material.

## Footnotes

^1^https://github.com/B-UMMI/INNUca

^2^https://github.com/tseemann/mlst

^3^http://pubmlst.org

^4^https://github.com/tseemann/abricate

^5^http://www.genomicepidemiology.org

^6^https://cge.food.dtu.dk/services/LRE-finder/

^7^https://github.com/ncbi/amr/wiki/Running-AMRFinderPlus

^8^https://cge.food.dtu.dk/services/VirulenceFinder/

^9^https://cge.food.dtu.dk/services/PlasmidFinder/

^10^https://github.com/B-UMMI/chewBBACA

^11^https://online2.phyloviz.net/index

^12^http://www.atgc-montpellier.fr/fastme/

^13^https://microreact.org/
